# Predictors of patient preference for either whole body magnetic resonance imaging (WB‐MRI) or CT/ PET‐CT for staging colorectal or lung cancer

**DOI:** 10.1111/1754-9485.13038

**Published:** 2020-05-14

**Authors:** Anne Miles, Ruth EC Evans, Steve Halligan, Sandy Beare, John Bridgewater, Vicky Goh, Sam M Janes, Neal Navani, Alfred Oliver, Alison Morton, Steve Morris, Andrea Rockall, Stuart A Taylor, the STREAMLINE investigators, A Aboagye, L Agoramoorthy, S Ahmed, A Amadi, G Anand, G Atkin, A Austria, S Ball, F Bazari, R Beable, H Beedham, T Beeston, N Bharwani, G Bhatnagar, A Bhowmik, L Blakeway, D Blunt, P Boavida, D Boisfer, D Breen, S Burke, R Butawan, Y Campbell, E Chang, D Chao, S Chukundah, B Collins, C Collins, V Conteh, J Couture, J Crosbie, H Curtis, A Daniel, L Davis, K Desai, M Duggan, S Ellis, C Elton, A Engledow, C Everitt, S Ferdous, A Frow, M Furneaux, N Gibbons, R Glynne‐Jones, A Gogbashian, S Gourtsoyianni, A Green, Laura Green, Liz Green, A Groves, A Guthrie, E Hadley, A Hameeduddin, G Hanid, S Hans, B Hans, A Higginson, L Honeyfield, H Hughes, J Hughes, L Hurl, E Isaac, M Jackson, A Jalloh, R Jannapureddy, A Jayme, A Johnson, E Johnson, P Julka, J Kalasthry, E Karapanagiotou, S Karp, C Kay, J Kellaway, S Khan, D Koh, T Light, P Limbu, S Lock, I Locke, T Loke, A Lowe, N Lucas, S Maheswaran, S Mallett, E Marwood, J McGowan, F Mckirdy, T Mills‐Baldock, T Moon, V Morgan, S Nasseri, P Nichols, C Norman, E Ntala, A Nunes, A Obichere, J O'Donohue, I Olaleye, A Onajobi, T O'Shaughnessy, A Padhani, H Pardoe, W Partridge, U Patel, K Perry, W Piga, D Prezzi, K Prior, S Punwani, J Pyers, H Rafiee, F Rahman, I Rajanpandian, S Ramesh, S Raouf, K Reczko, A Reinhardt, D Robinson, P Russell, K Sargus, E Scurr, K Shahabuddin, A Sharp, B Shepherd, K Shiu, H Sidhu, I Simcock, C Simeon, A Smith, D Smith, D Snell, J Spence, R Srirajaskanthan, V Stachini, S Stegner, J Stirling, N Strickland, K Tarver, J Teague, M Thaha, M Train, S Tulmuntaha, N Tunariu, K van Ree, A Verjee, C Wanstall, S Weir, S Wijeyekoon, J Wilson, S Wilson, T Win, L Woodrow, D Yu

**Affiliations:** ^1^ Department of Psychological Sciences Birkbeck University of London London UK; ^2^ Centre for Medical Imaging University College London Charles Bell House UK; ^3^ Cancer Research UK University College London Clinical Trials Centre London UK; ^4^ UCL Cancer Institute Paul O Gorman Building London UK; ^5^ Cancer Imaging School of Biomedical Engineering and Imaging Sciences King’s College London Strand, London UK; ^6^ Lungs for Living Research Centre UCL Respiratory Division of Medicine University College London London UK; ^7^ Department of Thoracic Medicine UCLH and Lungs for Living Research Centre UCL Respiratory University College London London UK; ^8^ Cancer patient representatives c/o National Cancer Research Institute London UK; ^9^ Research Department of Applied Health Research University College London London UK; ^10^ Department of Surgery and Cancer Imperial College London Kensington, London UK

**Keywords:** cancer, magnetic resonance imaging, patient preference, positron emission tomography, tomography, X‐ray computed

## Abstract

**Introduction:**

Whole body magnetic resonance imaging (WB‐MRI) may be more efficient in staging cancers, but can be harder for patients to tolerate. We examined predictors of patient preference for WB‐MRI vs. CT/ PET‐CT for staging colorectal or lung cancer.

**Methods:**

Patients recruited prospectively to two multicentre trials comparing diagnostic accuracy of WB‐MRI with standard staging scans were sent two questionnaires: the first, administered at trial registration, captured demographics, educational level and comorbidities; the second, administered after staging completion, measured emotional distress (GHQ‐12), positive mood (PANAS), perceived scan burden, patients’ beliefs about WB‐MRI, and preference for either WB‐MRI or CT (colorectal trial), WB‐MRI or PET‐CT (lung trial). Preference for WB‐MRI or CT/ PET‐CT was analysed using logistic regression.

**Results:**

Baseline and post‐staging questionnaires were completed by 97 and 107 patients, respectively. Overall, 56/107 (52%) preferred WB‐MRI over standard scans and were more likely to have no additional comorbidities, higher positive mood, greater awareness of potential benefits of WB‐MRI and lower levels of perceived WB‐MRI scan burden. In adjusted analyses, only awareness of potential WB‐MRI benefits remained a significant predictor (OR: 1.516, 95% CIs 1.006–2.284, *P* = 0.047). Knowledge that WB‐MRI does not use radiation predicted preference (adjusted OR: 3.018, 95% CIs 1.099–8.288, *P* = 0.032), although only 45/107 (42%) patients were aware of this attribute.

**Conclusions:**

A small majority of patients undergoing staging of colorectal or lung cancer prefer WB‐MRI to CT/ PET‐CT. Raising awareness of the potential benefits of WB‐MRI, notably lack of ionizing radiation, could influence preference.

## Introduction

Optimal management of patients diagnosed with cancer relies upon efficient and accurate staging, in particular, identification of metastatic disease. Current staging pathways are often complex, time consuming and involve several imaging modalities, most of which use ionizing radiation that has been linked to radiation‐induced malignancies, particularly in those exposed when young.^1^ Whole body magnetic resonance imaging (WB‐MRI) is an emerging technology that can image the body in less than one hour without ionizing radiation and could improve staging efficiency by reducing need for multiple scans, whilst at least matching diagnostic accuracy for metastases.^2^ WB‐MRI is already employed routinely for staging certain cancers, notably multiple myeloma.^3^ Recently, two large multicentre prospective trials found that accuracy of WB‐MRI staging pathways in colorectal and lung cancer does not differ significantly from standard pathways, but reduces time to staging, the number of tests required and overall costs.^4^, ^5^

Evaluation of new healthcare technology must fully consider the preferences, needs and values of the patient^6^ which in the case of WB‐MRI means comparison with standard pathways.^7^ A single staging WB‐MRI could reduce the psychological burden of prolonged and intensive multi‐modality staging^8^ whilst avoiding radiation exposure.^1^ Conversely, WB‐MRI can be stressful: scan time exceeds standard alternatives and MRI scanners are noisy, and require full body and head immersion inside a relatively narrow ‘tube’. Somewhere between 4 and 30% of patients experience some distress before and during MRI.^9^ Indeed, recent data show patients experience greater psychological and physical ‘burden’ during WB‐MRI than either CT or PET‐CT, and perceived burden is increased in those with high levels of emotional distress and existing medical comorbidities.^10^ The extent to which this influences patient preference is, however, unknown; particularly in patients aware of the potential benefits of WB‐MRI.

Factors underpinning patient cancer staging preferences are complex. Patients rate rapid diagnosis and treatment as one of the most important aspects of hospital‐based care,^11^ but are prepared to wait for results if they are more accurate.^12^ To date, most imaging preference studies have concentrated on the physical aspects of scanning experience, without examining patients’ understanding of other attributes, such as diagnostic accuracy and safety. For example, a recent study suggested breast cancer patients preferred spectral mammography over breast MRI,^13^ yet patients were told to assume both scans were of equivalent diagnostic accuracy and there was no assessment of attitudes towards, or awareness of, ionizing radiation exposure.

The present study aimed to identify predictors of patient preferences for either WB‐MRI or CT/ PET‐CT when staging known or highly suspected colorectal or lung cancer.

## Methods

Patients recruited to two parallel multicentre clinical trials comparing the diagnostic accuracy and cost‐effectiveness of WB‐MRI with standard tests for staging colorectal (Streamline C) and lung cancer (Streamline L) were invited to complete postal questionnaires before and after staging. Full ethical permission was given by Camden and Islington National Research Ethics Service (NRES) on 03/10/2012, project numbers: 12/LO/1176 (Streamline C) and 12/LO/1177 (Streamline L). The full protocols can be found at,^14^ but, in summary, eligible patients had known or suspected colorectal or lung cancer and were due to undergo staging. As part of the main trials, patients underwent WB‐MRI in addition to all standard staging tests.

Trial informed consent procedure involved patients receiving, prior to participation, an information sheet detailing the trial protocol and research interventions, which described the trial rationale and detailed potential benefits of WB‐MRI (see Appendix S1). Patients also received standard CT and PET‐CT information sheets as appropriate from their local recruitment site, given these tests were performed as part of routine clinical care.

Participants were recruited to the Streamline trials from 22 hospitals in the UK, and consented to participate in either interview or questionnaire studies to gauge their experience of staging and the influence of scan attributes on scan preference. The results of the interview study have been reported elsewhere,^15^ as have data from the present cohort pertaining to the perceived burden of WB‐MRI and standard staging scans,^10^ and a discrete choice experiment assessing the influence of scan attributes on patient preferences.^16^ This current report pertains to patient preferences for WB‐MRI and standard scans, along with predictors of preferences.

### Questionnaires

Baseline questionnaires were mailed to patients within 1 to 2 days after consenting to participate and whilst they were still undergoing staging. The post‐staging questionnaire was sent one month after the baseline questionnaire, when staging was complete. In the covering letter for the post‐staging questionnaire, patients were encouraged to return the baseline questionnaire if they had not yet done so. Patients were provided with stamped addressed reply envelopes and were paid £20 upon receipt of two completed questionnaires.

Consecutive patients were approached to participate until a minimum of 100 patients had returned the post‐staging questionnaire. The study was originally powered to assess the comparative patient‐perceived burden of WB‐MRI and standard staging tests, as reported previously.^10^

For patients with suspected or known colorectal cancer recruited to Streamline C, questionnaires referred to CT of the chest, abdomen and pelvis (the standard staging scan for colorectal cancer) and WB‐MRI. For patients with known or suspected non‐small cell lung cancer recruited to Streamline L, questionnaires referred to whole body PET‐CT (the standard staging scan for lung cancer) and WB‐MRI.

### Baseline questionnaire content

#### Demographics

Patient age and gender were collected; missing data were populated using registration information held at the clinical trial centre (with patient consent). Educational level was assessed using the question from the 2011 Census for England with the addition of a ‘prefer not to say’ response option. Post/zip code data were used to calculate an area‐based deprivation score for each individual using the 2010 IMD scale^17^ which was then categorized into quintiles with 1 representing highest levels of deprivation and 5 lowest.

### Follow‐up questionnaire content

#### Comorbidities

Patients were asked to report (‘yes’ or ‘no’) whether they had any of the following diseases: heart or vascular disease, diabetes, epilepsy, stroke, arthritis, asthma, mental or emotional disorder. There was also an option to provide details of any other illness.

#### Emotional distress

Distress was assessed using the 12 item General Health Questionnaire (GHQ‐12),^18^, ^19^ which asks patients to compare their psychological state over the last three months with their normal functioning level and includes items related to anxiety, happiness, depression, decision making, confidence, concentration and sleep disturbance. Six items are framed positively, with four response options ranging from ‘better/more than usual’ to ‘much less than usual’ and six items framed negatively with four responses options ranging from ‘not at all’ to ‘much more than usual’. An example item is as follows: ‘In the last three months have you….felt you couldn’t overcome your difficulties’.

#### Positive mood

Current mood was assessed by the positive subscale of PANAS, a 10 item scale, where positive affect is described as ‘a state of high energy, full concentration and pleasurable engagement’.^20^, ^21^

#### Beliefs regarding potential benefits of WB‐MRI

Patient perceptions regarding potential benefits of WB‐MRI compared to standard staging were assessed using five items developed specifically for the study. The first 2 items were factual and related to use of ionzing radiation, or otherwise, by the tests. ‘The Whole Body MRI scan uses X‐ray radiation’. (correct answer = No) ‘The CT [PET‐CT] scan uses X‐ray radiation’. (correct answer = Yes). The final 3 items referred to beliefs regarding potential benefits of WB‐MRI that had not yet been fully established: ‘If doctors use a Whole Body MRI scan, patients might need fewer scans in total to diagnose and stage their cancer’. ‘If doctors use a Whole Body MRI scan, patients might know sooner what their full diagnosis is (i.e. not just whether they have cancer, but whether their cancer has spread)’. ‘The whole body MRI scan is more accurate than CT [PET‐CT] at detecting cancer and discovering whether cancer has spread’. Response options given were ‘yes’, ‘no’ and ‘not sure’. Responses were summed to generate a total score ranging from 0 to 5, where 5 equates to more positive beliefs regarding WB‐MRI.

#### WB‐MRI scan burden

Patients were asked to document their experience of WB‐MRI using a 26‐item scale modified from an instrument developed to assess experience of colonoscopy.^22^ The scale has previously been adapted to better capture experience of diagnostic imaging scans^23^ and for the current study was modified further to include items deemed relevant by the researchers to WB‐MRI, based on interview data from initial patients recruited. The scale had three domains: scan discomfort (13 items), worry (6 items) and satisfaction (7 items). Response options were on a 1 to 7 scale, anchored at both ends, with participants asked to indicate experience by ticking along the scale. An example discomfort item was 1=’claustrophobic’ to 7= ‘not claustrophobic’. Questionnaire content and results from the current study cohort has been reported previously.^10^

#### Scan preference

Patients were asked: ‘If you had to have JUST ONE of the tests again which one would you prefer? Please circle or underline the one you would choose’. Choices given were WB‐MRI and CT (Steamline C), and WB‐MRI and PET‐CT (Streamline L).

### Statistical analysis

Data were analysed using SPSS version 24. Demographics of responders and non‐responders, and of participants recruited to Streamline C and Streamline L, were analysed using independent t‐tests for continuous variables and chi‐square for categorical variables.

Logistic regression was used to identify predictors of scan preference (WB‐MRI vs CT/PET‐CT). Predictors (age, gender, educational qualifications, cancer type, presence of comorbidities, emotional distress, positive mood, beliefs about WB‐MRI scans and WB‐MRI scan burden) were entered individually in an unadjusted analysis, and those that were significant or approaching significance (*P* < 0.10) were entered into a final adjusted analysis. Spearman’s rank correlation coefficients were computed for all variables entered into the adjusted regression model.

The 5 scores pertaining to beliefs regarding WB‐MRI were summed (range 0 to 5) and also tested separately to assess influence of specific beliefs on scan preference. Educational level was categorized into ‘some’ vs ‘no’ qualifications; comorbidity responses were summed to form a dichotomous variable (any comorbidity ‘present’ versus ‘absent’) removing emotional distress due to overlap with the GHQ‐12. The GHQ‐12 binary coding method (0‐0‐1‐1) was applied to each item, and total scores ranged from 0 to 12; a score of 4 or higher was used to indicate significant distress.^24^ Mean scan burden discomfort, worry and satisfaction domain scores were computed if at least 50% of component items were completed (for 7, 3 and 4 items, respectively). Internal reliability of subscales was adequate (Cronbach α ranged from 0.79 to 0.98). If less than 50% items were completed, the response was coded as missing. A total ‘Scan Burden’ score was calculated by taking the mean of discomfort, worry and (reverse scored) satisfaction subscales, with higher scores equating to greater burden.

## Results

### Response rates

**Fig. 1:**
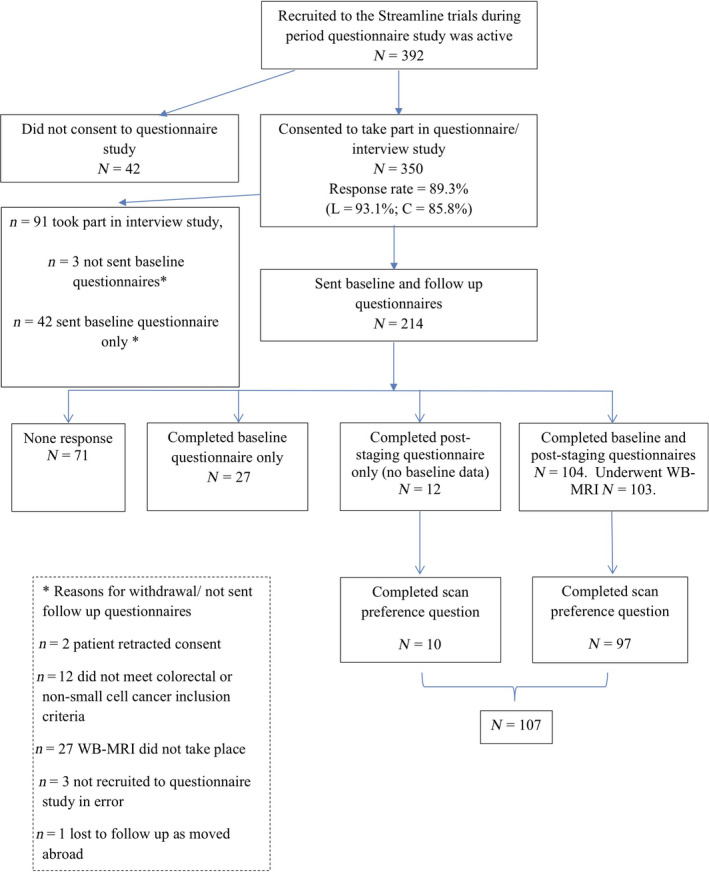
Flow diagram of participants through the study (March 2013–July 2015).

During the study period (March 2013 and July 2015), 392 people were recruited to the Streamline trials of whom 350 (89.3%) agreed to participate in the questionnaire or interview study (see Fig. 1). Ninety‐one were recruited to the interview study,^15^ and 45 were not sent both questionnaires. Of the remaining 214 patients sent both questionnaires, 71 did not respond. The final study cohort was 107 (defined as those completing the follow‐up questionnaire and answering the question on scan preference). Of this final cohort, 97 also completed the baseline questionnaire.

There were no significant differences between the final study cohort and those who were sent both questionnaires but did not complete the scan preference question for age (t < 1; df = 212; *P* = 0.746), cancer type [colorectal or lung] (chi‐square = 1.209; df = 1; *P* = 0.272) or deprivation (chi‐square = 4.261; df = 2; *P* = 0.119). However, women were more likely to complete questionnaires than men (59.7% vs 44.5%; chi‐square = 4.564; df = 1; *P* = 0.033).

### Demographics

Average participant age was 64.7 years (range: 30 to 88), with no differences between men and women (average age and range: 65.1 (30 to 85) vs. 64.2 (35 to 88), respectively; t < 1, df = 105, *P* = 0.679). Sixty‐two (58%) were male, 62 (72%) had educational qualifications, and 55 (59%) reported at least one additional comorbidity (see Table 1).

**Table 1:** Predictors of scan preference for WB‐MRI compared with CT/PET‐CT

Predictor	Sample characteristics	Odds Ratios/ Exp_B (CI)	
	(% and *N* unless otherwise specified)	Unadjusted (*N* = 107)	Adjusted (*N* = 83)
Demographic and clinical variables			
Age^†^	64.7 (11.9) (mean, SD)	0.969 [0.937 to 1.002] *P* = 0.067	0.976 [0.927 to 1.028]
Gender^†^			
Female	42.1 (45)	[1.00]	
Male	57.9 (62)	1.269 [0.588 to 2.740]	
Educational qualifications^§^			
No	27.9 (24)	[1.00]	[1.00]
Yes	72.1 (62)	2.593 [0.967 to 6.950] *P* = 0.058	1.750 [0.498 to 6.145]
Cancer type^†^			
Colorectal	51.4 (55)	[1.00]	‐
Lung	48.6 (52)	0.616 [0.287 to 1.323]	
Physical and emotional wellbeing			
Presence of comorbidities ^§^			
No	41.5 (39)	[1.00]	[1.00]
Yes	58.5 (55)	0.299 [0.128 to 0.698] *P* = 0.005	0.498 [0.170 to 1.453]
Emotional distress (GHQ‐12, post‐staging)^†^			
No	58.3 (56)	[1.00]	[1.00]
Yes	41.7 (40)	0.494 [0.227 to 1.072] *P* = 0.074	1.135 [0.381 to 3.386]
Positive mood (PANAS, post‐staging)^‡^	28.37 (8.82) (mean, SD)	1.059 [1.009 to 1.111] *P* = 0.020	1.044 [0.979 to 1.113]
Scan beliefs and experience			
Beliefs about potential benefits of WB‐MRI^†^ (total score)	3.06 (1.30) (mean, SD)	1.489 [1.082 to 2.049] *P* = 0.015	1.516 [1.006 to 2.284] *P* = 0.047
Total WB‐MRI patient burden score^‡^	2.24 (1.07) (mean, SD)	0.658 [0.439 to 0.984] *P* = 0.042	0.648 [0.362 to 1.161]

^†^ No missing data.

^‡^ Missing data less than 5%.

^§^ Missing data greater than 5%. Where there is missing data per cent is valid per cent.

Patients recruited to Streamline C were younger (61.7 vs. 67.9: t = 2.75; df = 105; *P* = 0.007), more likely to have educational qualifications (83% vs. 60%; chi‐square = 5.44; df = 1; *P* = 0.020), less likely to report comorbidities (44% vs. 69%; chi‐square = 6.49; df = 1; *P* = 0.011) than patients recruited to Streamline L. There were no differences between Streamline C and Streamline L cohorts regarding gender (% male 60 vs. 56 respectively; chi‐square = 0.196; df = 1; *P* = 0.658), levels of positive mood post‐staging (29.0 vs. 26.7, respectively; t = 1.352; df = 103; *P* = 0.179) and presence of emotional distress (% distressed 47 vs. 40, respectively; chi‐square = 0.515; df = 1; *P* = 0.473).

#### Beliefs regarding potential benefits of WB‐MRI

Ninety‐one (85%) patients believed that WB‐MRI might lead to more rapid diagnosis and staging, 80 (75%) that WB‐MRI might lead to fewer scans, and 52 (49%) that WB‐MRI is more accurate than CT/PET‐CT. However, just 45/107 (42%) were aware that WB‐MRI did not use ionizing radiation, with 59 (55%) aware that CT/PET‐CT did.

Patients recruited to Streamline C had, in general, more positive beliefs regarding WB‐MRI than those recruited to Streamline L (3.42 vs. 2.67; *t* = 3.08, df = 105, *P* = 0.003), although when belief items were tested individually, only perceptions regarding WB‐MRI accuracy were significantly different between the two cohorts; 36 (66%) of patients recruited to Streamline C believed WB‐MRI was more accurate than CT compared to 16 (31%) of patients recruited to Streamline L, who thought WB‐MRI more accurate than PET‐CT (chi‐square = 12.873, df = 1; *P* < 0.001).

#### Scan preference

Overall, 56/107 (52%) patients expressed a preference for WB‐MRI over standard tests which, in the unadjusted regression analysis, was independent of cancer type or gender (Table 1). Patients with higher positive mood scores, no comorbidities, greater awareness of potential benefits of WB‐MRI and lower total perceived burden scores for WB‐MRI were more likely to express preference for WB‐MRI, with younger age, educational qualifications and absence of high emotion distress approaching significance (Table 1).

In adjusted analyses, only greater belief in benefits of WB‐MRI (OR: 1.516, 95% CIs 1.006 to 2.284, *P* = 0.047) remained a significant predictor of patient preference for WB‐MRI. Of the 5 items pertaining to the potential benefits of WB‐MRI, only awareness that WB‐MRI does not involve radiation predicted patient preference (unadjusted OR: 2.350; 95% CIs: 1.066 to 5.179; *P* = 0.034; adjusted OR: 3.018, 95% CIs 1.099 to 8.288, *P* = 0.032) (see Table A1: Appendix S2). Just over sixty‐four per cent of people who were aware WB‐MRI did not use radiation preferred WB‐MRI to standard scans (64.4%, *n* = 29/45), compared with 43.5% of people unaware of this attribute (*n* = 27/62). Correlations between variables entered into the regression model are shown in Table 2.

**Table 2:** Correlations between predictors of scan preference entered in the adjusted regression analysis

	Age	Educational qualifications	Comorbidity	Positive mood	Emotional distress	WB‐MRI scan beliefs
Educational qualifications	−0.465	–				
	*P* < 0.001					
	*n* = 86					
Comorbidity	0.268	−0.122	–			
	*P* = 0.008	*n* = 86				
	*n* = 97					
Positive mood	0.148	−0.031	−0.251	–	–	
	*n* = 105	*n* = 84	*P* = 0.014			
			*n* = 95			
Emotional distress	−0.050	0.055	0.207	−0.366	–	–
	*n* = 107	*n* = 86	*P* = 0.042	*P* < 0.001		
			*n* = 97	*n* = 105		
WB‐MRI scan beliefs	−0.005	0.244	−0.080	0.117	−0.136	
	*n* = 107	*P* = 0.023	*n* = 97	*n* = 105	*n* = 107	
		*n* = 86				
WB‐MRI scan burden	0.000	−0.010	0.236	−0.312	0.342	−0.102
	*n* = 106	*n* = 85	*P* = 0.020	*P* < 0.001	*P* < 0.001	*n* = 106
			*n* = 96	*n* = 104	*n* = 106	

## Discussion

We examined preferences for WB‐MRI compared to CT and PET‐CT amongst 107 patients with suspected or known lung or colorectal cancer. We found that a small majority expressed a preference for WB‐MRI, contrasting with previous data suggesting that, in general, patients prefer scans such as CT and PET‐CT over MRI,^13^, ^25^ even when using short‐bore MRI machines and rapid acquisition protocols.^13^

For example, Shortman et al.^25^ compared scan burden and preference amongst patients with a variety of medical conditions (including cancer) referred for PET‐CT who also volunteered to undergo PET‐MRI. Overall, participants reported greater burden during PET‐MRI and a higher proportion preferred PET‐CT (24.6% vs. 52.2%, respectively). Those expressing a preference for PET‐MRI reported lower levels of burden during this, suggesting preferences were influenced by their perception of the scan experience itself. Similarly, Hobbs et al.^13^ reported that breast cancer patients preferred contrast‐enhanced spectral mammography (CESM) to contrast‐enhanced short‐bore MRI (CEMRI), largely due to a more positive experience during the former; reasons given included shorter scan duration, greater comfort and lower noise associated, despite patients preferring the type of breast compression used for CEMRI. However, the literature is not uniform in suggesting MRI is more burdensome than other imaging techniques. Adams et al.^26^ reported that patients experienced less burden during WB‐MRI than CT, a fact attributed largely by the authors to the need for an intravenous line and administration of contrast medium during CT.

In our cohort, we have already reported that, in general, patients experience greater burden during WB‐MRI than either CT or PET‐CT, although absolute differences in burden scores were small.^10^ In contrast to Adams et al.,^26^ the Streamline trials WB‐MRI protocol required intravenous contrast.

Despite the greater perceived patient burden of WB‐MRI, our data suggest that reasons underlying patients’ preferences are more complex than simply perception of the scan itself. Although, in our unadjusted analysis, greater perceived scan burden predicted scan preference, this was no longer the case in the adjusted model. Instead, the only predictor was greater belief in benefits of WB‐MRI. The trial material provided to patients described some potential benefits of MRI, including potentially reduced scan number and radiation exposure. Our data suggest therefore that patients can ‘trade’ discomfort experienced during WB‐MRI against positive beliefs regarding benefit. Indeed, using a discrete choice experiment, we have shown that most patients prefer WB‐MRI‐based staging pathways that are quicker, require fewer scans and avoid radiation, with 72% of colorectal cancer patients and 82% of patients with suspected lung cancer preferring such a pathway.^16^ The Streamline trails found WB‐MRI staging pathways possess these attributes over standard staging.^4^, ^5^

Interestingly, of the five potential benefits presented to patients in our questionnaire, only lack of radiation independently predicted preference, despite only 42% of patients being apparently aware of this. Previous research suggests patients’ knowledge regarding ionizing radiation amongst commonly used imaging technologies is relatively poor,^27^, ^28^ and once informed, patients are less willing to undergo scans such as CT.^29^ Indeed, the International Atomic Energy Agency state that medical radiation should consider patient concerns^30^ and it is known that cancer patients desire information regarding use of ionizing radiation for medical imaging.^27^ Should WB‐MRI disseminate in the NHS, radiation avoidance should be emphasized to patients given its influence on their preferences.

### Study limitations

During our study, the diagnostic accuracy outcomes of the Streamline trials were unknown, meaning that any potential advantages over standard scans, other than avoiding radiation, remained theoretical. It is therefore possible that preferences may change now the benefits of WB‐MRI are  established.^4^, ^5^ However, we aimed to identify which facets of WB‐MRI influence preference, and we were able to demonstrate that patients utilize perceived benefits of new technology in their preference decisions.

Our participants were, on average, younger than those typically diagnosed with lung or colorectal cancer and, by definition, they opted into a trial which required an additional WB‐MRI; patients with an existing fear of MRI will likely have declined participation. Our cohort may therefore not be truly representative of those referred for WB‐MRI in general.

We cannot be sure all patients read information sheets regarding WB‐MRI, and lack of ionizing radiation was mentioned only once, so could have been better emphasized.

In line with similar studies, questionnaire completion was higher amongst women and male preferences are underrepresented as a consequence. Additionally, adjusted analyses were underpowered and factors emerging as significant only in unadjusted analyses may nevertheless be important predictors of preferences.

### Conclusions

Of patients being staged for suspected or known lung or colorectal cancer, a small majority express preference for WB‐MRI over CT and PET‐CT. Greater belief in the benefits of WB‐MRI influences preference more than perceived scan burden. Lack of ionizing radiation exposure, in particular, influences preference for MRI, and patients should be fully informed of all staging pathway attributes.

## Supporting information

**Appendix S1.** Patient information sheet: sections detailing the rationale for the trial and potential benefits of WB‐MRI.
**Appendix S2.** Table A1: Predictors of scan preference for WB‐MRI compared with CT/PET‐CT with scan beliefs entered as individual items rather than a composite score.
