# Supplementary material for: Predictors of patient preference for either whole body magnetic resonance imaging (WB‐MRI) or CT/ PET‐CT for staging colorectal or lung cancer
Source: J Med Imaging Radiat Oncol. 2020 May 14;64(4):537–45. doi: 10.1111/1754-9485.13038 (PMC8425331; doi:10.1111/1754-9485.13038)
Supplement: Supplementary file 1 — Appendix S1. Patient information sheet: sections detailing the rationale for the trial and potential benefits of WB‐MRI. Appendix S2. Table A1: Predictors of scan preference for WB‐MRI compared with CT/PET‐CT with scan beliefs entered as individual items rather than a composite score. [file ARA-64-537-s001.docx]

**Supplementary material**

**Appendix A:**

Patient information sheet: sections detailing the rationale for the trial and potential benefits of WB-MRI.

The information sheet contained the following about the WB-MRI scan: “The reason for doing this trial is to investigate whether whole body magnetic resonance imaging (WB-MRI) is better or quicker at staging newly diagnosed cancer than the standard tests currently used. Cancer staging describes how far a cancer has spread from the main tumour to other parts of the body. Correct staging is important since doctors use this information to decide on treatment…. WB-MRI is a scanning technique which can image the whole body in about 1 hour. MRI scanning has been around for a while and is already commonly performed in hospitals. However, using MRI to look at the whole body in one go is quite new. The main advantage of WB-MRI may be for those patients with newly diagnosed or suspected cancer. Currently patients often undergo several different scans and tests which may be time consuming and involve multiple visits to the hospital. We want to see if one WB-MRI could replace the multiple scans and tests. … MRI is a safe test and does not use X-ray radiation… As part of your normal care you may undergo CT scans or PET/CT scans and they carry a possible risk as they involve exposure to radiation. It is important to remember that you would have had these scans in any case as part of your normal care and not because you agreed to take part in the trial. No additional scans using radiation are performed as part of the trial itself… It is possible WB-MRI may provide doctors with extra useful information about your condition which could help in your treatment, but we cannot guarantee this.”

**Appendix B:**

Table A1: Predictors of scan preference for WB-MRI compared with CT/PET-CT with scan beliefs entered as individual items rather than a composite score

| Predictor | Odds Ratios/ Exp_B (CI) | |
| --- | --- | --- |
|  | Unadjusted  (N=107) | Adjusted  (N=83) |
| *Demographic and clinical variables* |  |  |
| Age ^a^ | 0.969 [0.937 to 1.002]  p=0.067 | 0.980 [0.929 to 1.033] |
| Gender ^a^ |  |  |
| Female | [1.00] | - |
| Male | 1.269 [0.588 to 2.740] |  |
| Educational qualifications |  |  |
| No | [1.00] | [1.00] |
| Yes | 2.593 [0.967 to 6.950]  p=0.058 | 1.885 [0.528 to 6.734] |
| Cancer type ^a^ |  |  |
| Colorectal | [1.00] | - |
| Lung | 0.616 [0.287 to 1.323] |  |
| *Physical and emotional wellbeing* |  |  |
| Presence of comorbidities ^c^ |  |  |
| No | [1.00] | [1.00] |
| Yes | 0.299 [0.128 to 0.698]  p=0.005 | 0.591 [0.198 to 1.762] |
| Emotional distress (GHQ-12, post-staging) ^a^ |  |  |
| No | [1.00] | [1.00] |
| Yes | 0.494 [0.227 to 1.072]  p=0.074 | 0.878 [0.291 to 2.648] |
| Positive mood  (PANAS, post-staging) ^b^ | 1.059 [1.009 to 1.111]  p=0.020 | 1.036 [0.973 to 1.104] |
| *Scan experience* |  |  |
| Total WB-MRI patient burden score ^b^ | 0.658 [0.439 to 0.984]  p=0.042 | 0.635 [0.348 to 1.159] |
| *Scan beliefs* |  |  |
| WB-MRI scan uses x-ray radiation ^a^ |  |  |
| Yes or not sure | [1.00] | [1.00] |
| No | 2.350 [1.066 to 5.179]  p=0.034 | 3.018 [1.099 to 8.288]  p=0.032 |
| CT / PET-CT scan uses x-ray radiation ^a^ |  |  |
| No or not sure | [1.00] | - |
| Yes | 1.875 [0.867 to 4.055] |  |
| Use of WBMRI might lead to fewer scans ^a^ |  |  |
| No or not sure | [1.00] | - |
| Yes | 1.252 [0.522 to 2.998] |  |
| Use of WBMRI scan might lead to more rapid diagnosis and staging ^a^ |  |  |
| No or not sure | [1.00] | [1.00] |
| Yes | 2.805 [0.901 to 8.729]  p=0.075 | 1.175 [0.266 to 5.183] |
| WBMRI scan might be more accurate than CT / PET-CT at diagnosis and staging ^a^ |  |  |
| No or not sure | [1.00] | - |
| Yes | 1.521 [0.709 to 3.264] |  |

^a^ No missing data

^b^ Missing data less than 5%

^c^ Missing data greater than 5%
